# Partners’ childbirth experiences during the COVID-19 pandemic – findings from the Swedish COPE prospective cohort study

**DOI:** 10.1186/s12884-026-08862-3

**Published:** 2026-02-27

**Authors:** Eva Uhlander, Sara Isell, Sofie Arnkil, Johanna Berg, Gustaf Biasoletto, Marie Blomberg, Ylva Carlsson, Linda Hjertberg, Anna Sand, Anna-Karin Wikström, Mehreen Zaigham, Verena Sengpiel, Karolina Linden

**Affiliations:** 1 Institute of Health and Care Sciences, Sahlgrenska Academy, University of Gothenburg, Box 457, Gothenburg, 405 30 Sweden; 2 Department of Obstetrics and Gynecology, Region Jönköping County, Ryhov County Hospital, Jönköping, Sweden; 3 Department of Obstetrics and Gynecology, Region Skåne, Skåne University Hospital, Lund, Sweden; 4 Region Västra Götaland, Sahlgrenska University Hospital, Department of Obstetrics and Gynecology, Gothenburg, Sweden; 5 Department of Obstetrics and Gynecology, Institute of Clinical Sciences, Sahlgrenska Academy, University of Gothenburg, Gothenburg, Sweden; 6 Department of Surgery and Orthopedics, Region Västra Götaland, Högsbo Hospital, SV Hospital Group, Gynecology Clinic, Gothenburg, Sweden; 7 Department of Women’s Health, Region Jämtland Härjedalen, Östersund Hospital, Östersund, Sweden; 8 Department of Obstetrics and Gynecology and Department of Biomedical and Clinical Sciences, Linköping University, Linköping, Sweden; 9 Centre of Perinatal Medicine & Health, Institute of Clinical Sciences, Sahlgrenska Academy, University of Gothenburg, Gothenburg, Sweden; 10 Region Östergötland, Vrinnevisjukhuset, Department of Obstetrics and Gynecology, Norrköping, Sweden; 11 Department of Biomedical and Clinical Sciences, Linköping University, Linköping, Sweden; 12 Department of Women’s and Children’s Health, Karolinska Institutet, Stockholm, Sweden; 13 Region Stockholm, Karolinska University Hospital, Division of Pregnancy and Childbirth, Department of Women’s Health, Stockholm, Sweden; 14 Department of Women’s and Children’s Health, Uppsala University, Uppsala, Sweden; 15 Region Uppsala, Uppsala University Hospital, Department of Obstetrics and Gynecology, Uppsala, Sweden; 16 Obstetrics and Gynecology, Institution for Clinical Sciences Lund, Lund University, Lund, Sweden; 17 Department of Obstetrics and Gynecology, Region Skåne, Skåne University Hospital, Malmö, Sweden

**Keywords:** Anxiety, Birth experience, COVID-19 pandemic, Depression, Father, Fathers for the first time questionnaire, Hospital anxiety and depression scale, Paternal mental health, Sense of coherence, Sweden

## Abstract

**Background:**

The COVID-19 pandemic disrupted maternity care and limited partner involvement during childbirth. While partners play an important role during pregnancy and birth, their own experiences remain understudied. This study aimed to measure partners’ childbirth experience during the COVID-19 pandemic and examine associations with relevant variables.

**Methods:**

This cross-sectional, prospective cohort study was part of the Swedish multicentre COVID-19 during Pregnancy and Early Childhood Study (COPE). Data were collected through electronic questionnaires and national health and quality registers. Childbirth experience was measured using the Fathers for the First Time Questionnaire (FTFQ), comprising of four dimensions: ‘Worry’ (concerns about safety and emotional reactions), ‘Information’ (perceived adequacy and relevance of information), ‘Acceptance’ (feeling welcomed and acknowledged by staff), and ‘Emotional support’ (experiences of guidance and reassurance during birth). Hierarchical multiple linear regression was used to examine associations with selected variables.

**Results:**

A total of 365 partners (54.5% first-time parents) were recruited between June 2020 and December 2022. Mean (SD) FTFQ scores (range 0–4, lower scores indicate a more positive experience) were: ‘Worry’ 2.1 (0.7), ‘Information’ 1.7 (0.5), ‘Acceptance’ 1.3 (0.4), and ‘Emotional support’ 2.1 (0.7). Partners who did not experience the birth as expected, were exposed to a complicated birth, or reported symptoms of anxiety and/or depression during pregnancy had more negative childbirth experiences, particularly regarding ‘Worry’ and ‘Information’.

**Conclusions:**

During the COVID-19 pandemic, partners who reported unmet expectations of childbirth, exposure to birth complications, or antenatal symptoms of anxiety and/or depression appeared to be particularly vulnerable to more negative childbirth experiences, especially in the dimensions ‘Worry’ and ‘Information’. These findings underscore the importance of identifying psychological vulnerability and unmet informational needs among partners during pregnancy and birth, and of fostering communication strategies that promote inclusion and emotional safety in the childbirth setting.

**Trial registration:**

NCTO4433364 06/02/2020.

**Supplementary Information:**

The online version contains supplementary material available at 10.1186/s12884-026-08862-3.

## Background

Partners of expectant women often experience pregnancy and childbirth as an emotional journey, characterised by both excitement and uncertainty [1]. Active engagement and involvement are central not only to prenatal bonding and the partner’s own childbirth experience, but also to their ability to provide effective support to the birthing woman during childbirth [1–3].

Partners commonly report a wide range of intense emotions in connection with childbirth. While the process may involve overwhelming feelings and fear, the moment of birth is often described as deeply meaningful and emotionally rewarding [3]. Although the birth itself may be remembered as a powerful and positive event, the overall experience can also entail significant emotional strain, particularly when complications occur or expectations are unmet. For birthing women, a negative birth experience has been associated with both immediate and long-term adverse outcomes, including an increased risk of postnatal depression, post-traumatic stress disorder, and fear of childbirth in subsequent pregnancies [4, 5]. How negative experiences affect partners remains insufficiently studied. However, high levels of psychological distress have been reported among partners exposed to adverse fetal, neonatal or maternal outcomes, with such distress potentially persisting for several months and resulting in long-lasting consequences [6].

The COVID-19 pandemic led to substantial changes in maternity care, including restrictions on visitors before, during and after childbirth [7]. For birthing women Nordic studies during the COVID-19 pandemic describe increased worry, reduced emotional support, and feelings of isolation related to rapidly changing maternity care routines and restrictions on companionship [8, 9]. One qualitative study focusing on women who gave birth while ill with COVID-19 found that enforced separation from the newborn, and communication challenges related to infection-control measures could negatively affect their sense of safety during labour and the early postpartum period [10]. These findings align with systematic reviews demonstrating that service reorganisation and restricted support sometimes undermined women’s overall childbirth experience in high-income settings [11, 12].

Partners, who typically value active involvement in pregnancy and childbirth as part of their preparation for parenthood [13], were significantly affected by these changes. Exclusion from maternity care settings contributed to a sense of disconnection and a loss of involvement in the transition to parenthood [14]. Partners reported feelings of isolation, psychological distress, and reduced opportunities for early bonding with their newborns [7, 14]. However, it is plausible that the intensity of the pandemic’s impact varied over time and was influenced by factors such as year of birth, vaccination status, and previous or ongoing infection with SARS-CoV-2.

For birthing women, a negative birth experience may be influenced by individual factors such as age, parity, fear of childbirth and level of preparation; interpersonal factors such as the degree of support received; and obstetric complications, including prolonged labour, instrumental birth, emergency caesarean section or poor neonatal outcomes [15]. Although less studied, similar interpersonal and obstetrical factors have been described in relation to partners’ experiences [16]. In contrast, individual-level determinants of negative birth experiences among partners remain insufficiently explored. Partners with a history of depression may be particularly vulnerable to negative perceptions of childbirth [17]. The prevalence of prenatal depression among partners (fathers) has been estimated at approximately 10 per cent, although rates vary across populations and settings [17]. Further, previous studies indicate that first-time and experienced partners may differ in their expectations, level of preparation and emotional responses to childbirth, partly due to prior childbirth experiences or previous traumatic events [18].

Despite growing awareness of partners’ emotional involvement in childbirth, few studies have examined which factors influence their experiences, particularly in the context of healthcare disruptions such as those caused by the COVID-19 pandemic. While previous research has reported general distress among partners during the pandemic, there is a lack of studies employing validated instruments to assess the multidimensional nature of partners’ childbirth experience. This study aims to measure partners’ childbirth experience during the COVID-19 pandemic and examine associations with relevant variables.

## Methods

### Study design

The current cross-sectional prospective cohort study is part of the Swedish multicentre COVID-19 during Pregnancy and Early Childhood (COPE) study (Clinical Trials Registration number NCT04433364) [19]. Participants were recruited between June 2020 and December 2022 through antenatal screening or at hospital birth units. Pregnant women could choose to participate in the questionnaire part, the biological analysis part, or both. Their partners were invited to take part in the questionnaire part of the study. The dataset was linked to national health and quality registers using the Swedish personal identification number.

### Study population

Eligible participants in the present study were individuals who identified as the partner, regardless of gender, of a pregnant woman enrolled in the COPE study. The study included both first-time and experienced partners, as childbirth experience was not restricted by parity in the present analysis. Inclusion criteria required participants to be over 18 years of age and proficient in either Swedish or English. Only partners who were present during a birth that commenced vaginally were eligible, regardless of whether the birth resulted in a normal vaginal birth (partus normalis), an instrumental birth or an emergency caesarean section as the applied questionnaire is not validated for women who did not undergo labour.

### Data collection

Participants in the COPE study who took part in the questionnaire part received electronic surveys at inclusion and during set timepoints post-partum. Reminders were sent out automatically [19]. The present study is based on data collected using validated instruments. Partners’ experiences of childbirth were assessed with the *Fathers for the First Time Questionnaire* (FTFQ) answered eight to twelve weeks postpartum [20]. Symptoms of anxiety and depression were measured using the *Hospital Anxiety and Depression Scale* (HADS) answered at inclusion into the COPE study (during pregnancy) [21], and global orientation towards life was assessed using the 13-item *Sense of Coherence scale* (SOC-13) answered during pregnancy [22].

### Research questions

This study was designed to address the following research questions:

1. How did partners experience childbirth during the COVID-19 pandemic as measured by the FTFQ and its dimensions (‘Worry’, ‘Information’, ‘Acceptance’, and ‘Emotional support’)?
2. Are sociodemographic determinants, global orientation towards life, and obstetric determinants associated with variations in partners’ experiences of childbirth during the COVID-19 pandemic, as measured by the FTFQ?
3. Are anxiety and/or depression, as measured by the Hospital Anxiety and Depression Scale (HADS) before childbirth, associated with the different dimensions of partners’ childbirth experience as measured by the FTFQ?

### Outcome definition

The FTFQ consists of 22 items divided into four subscales assessing different aspects of the childbirth experience: ‘Worry’ (eight items measuring concerns regarding the health and safety of the birthing woman and baby, uncertainty about one’s ability to provide support, personal emotional reactions, and fear of the unknown); ‘Information’ (four items assessing how well prepared the partner felt and whether they perceived that adequate and relevant information was provided during childbirth); ‘Acceptance’ (four items measuring whether the partner felt welcomed, respected, and acknowledged by healthcare professionals); and ‘Emotional support’ (six items assessing the extent to which partners experienced guidance, reassurance, and comfort from healthcare staff during the birth). Responses were rated on a four-point Likert scale: 1 = completely true, 2 = partly true, 3 = somewhat true, and 4 = not true at all. Negatively worded items were reverse-scored, and lower scores indicated a more positive childbirth experience [20]. The FTFQ was used regardless of parity, as the instrument assesses the childbirth experience of partners generally rather than exclusively first-time parents, as described in its validation [20]. It was also applied irrespective of the partner’s gender, based on the assumption that the non-birthing partner’s role and experience are comparable across genders. Internal consistency for the FTFQ subscales was assessed in the present sample. Cronbach’s alpha values for first-time partners were α = 0.83 (‘Worry’), α = 0.67 (‘Information’), α = 0.67 (Acceptance’), and α = 0.72 (‘Emotional support’). For experienced partners, alpha values were α = 0.86, α = 0.62, α = 0.62, and α = 0.76, respectively.

### Exposure definition

#### Research question 1

##### Birth as expected

Birth as expected was measured by the self-reported item: *“Did you experience the childbirth as you had expected?”*, to which participants responded with a binary answer (yes/no). The item is included in the FTFQ instrument but is not incorporated into the score calculations [20]. Based on these responses, participants were categorised into two groups: those who experienced the birth as expected and those who did not.

#### Research question 2

##### Sociodemographic determinants

Sociodemographic determinants included first-time parent status (partner), satisfaction with birth preparations, educational level of the partner, year of birth and parity of the birthing woman. First-time parent status, satisfaction with birth preparations (yes/no), and partner’s educational attainment (university level: yes/no) were based on self-reported questionnaire data answered either at inclusion or eight to twelve weeks postpartum. Parity of the birthing woman (primiparous/multiparous) and year of birth (categorised as 2020, 2021 or 2022) were obtained from the Swedish Pregnancy Register [23].

##### Global orientation towards life

Global orientation towards life was measured using the Sense of Coherence (SOC) scale answered at inclusion [22]. SOC reflects a person’s overall capacity to respond to stress in a health-promoting way and is positively associated with psychological well-being [24]. The SOC-13 scale consists of 13 items rated on a range from 1 to 7. Negatively worded items were reverse-scored, and a total score of 60 points or less was defined as indicating a low level of sense of coherence [25]. Participants who had filled out SOC on or after the birth date of the child have been excluded from the analyses (regarded as missing).

##### Obstetric determinants

Obstetric determinants included mode of labour onset (induced vs spontaneous). *Complicated birth* was defined as the presence of one or more of the following conditions: postpartum haemorrhage greater than 1000 ml, emergency caesarean section, instrumental vaginal birth, preterm birth (before 37 + 0 gestational weeks + days), NICU admission during the first 2 days following birth, Apgar score at 5 min below 4 or manual removal of the placenta. This information was obtained from the Swedish Pregnancy Register [23] as well as the Swedish neonatal quality register [26]. Please note, in Sweden, all infants requiring observation or treatment beyond routine postpartum care are recorded under “NICU admission” in the Swedish neonatal quality register. This category includes both high-dependency neonatal intensive care and lower-level special care units.

For the birthing woman, information on COVID-19 vaccination status at the time of birth was obtained from the National Vaccination Register [27], while data on confirmed COVID-19 infection during pregnancy and/or at the time of birth were retrieved from the Register of Notifiable Communicable Diseases [28] the National Patient Register [29] and the Swedish pregnancy register [23].

#### Research question 3

##### Symptoms of anxiety or depression

Symptoms of anxiety and depression were assessed using the *Hospital Anxiety and Depression Scale* (HADS) answered at inclusion [21]. Any participant who completed the questionnaire on or after the birth date was excluded from the analyses. The instrument comprises two self-reported subscales: one for anxiety and one for depressive symptoms. Each subscale was scored and categorised as follows: no symptoms (≤ 7), mild symptoms (8–10), and severe symptoms (> 10) [21, 30, 31]. In the present study, no distinction was made between mild and severe symptoms; instead, these categories were combined to indicate the presence of anxiety and/or depression. Given the common comorbidity between anxiety and depression, these constructs were analysed in combination [32]. Any HADS questionnaire completed on or after the child’s birth date was excluded from analyses addressing antenatal anxiety and depressive symptoms, to ensure that the measure reflected prenatal rather than postnatal mental health.

All other variables presented in the background found in Table 1, Demographic and baseline characteristics*,* were obtained from the following sources: maternal and obstetric variables retrieved from the Swedish Pregnancy Register [23] included parity and age of the birthing woman, her relation to the participant (her partner) year of childbirth, onset of labour, mode of birth, gestational week at birth, and use of in vitro fertilisation. Additional clinical indicators obtained from the Swedish Pregnancy Register were postpartum haemorrhage exceeding 1000 ml, manual removal of the placenta, Apgar score below 7 at 5 min, and preterm birth (less than 37 gestational weeks). For the birthing woman information on COVID-19 vaccination status was obtained from the National Vaccination Register [27], and diagnoses of notifiable communicable diseases were collected from the Swedish Register of Notifiable Communicable Diseases [28]. Neonatal outcomes, including immediate admission to a neonatal intensive care unit, were obtained from the Swedish Neonatal Quality Register [26]. Demographic data were collected within the COPE study, either at study inclusion or at the eight-week postpartum follow-up.

**Table 1:** Demographic and baseline characteristics

Variable	*n* = 365	%
Parity partner		
First time parent	199	54.5
Parity birthing woman		
Primipara	199	54.5
Age partner (years)		
21–30	97	26.7
31–40	236	65.0
> 41	30	8.3
Missing	< 5	
Age birthing woman (years)		
21–30	126	35.5
31–40	231	63.3
> 41	8	2.2
Gender partner		
Male	359	98.4
Female	< 5	
Missing	< 5	
Studies at University level		
Yes	267	73.2
Missing	< 5	
Country of birth, partner		
Sweden	326	89.3
Europe	22	6
Outside Europe	17	4.7
Relationship to birthing woman		
Living together	344	94.2
Single living	< 5	
Other	< 5	
Missing	17	4.7
Satisfied with preparations before birth		
Yes	305	83.6
Type of preparation before birth (Several answers possible)		
Antenatal class	101	27.7
Self-sought information	276	75.6
Other preparation	55	15.1
No preparation	46	12.6
Low SOC		
Yes	49	13.4
No	138	37.8
Not replied to SOC	178	48.8
Year of childbirth		
2020	93	25.5
2021	200	54.8
2022	72	19.7
Vaccinated before birth, birthing woman		
Yes	114	31.2
COVID-19 status, birthing woman		
Positive during pregnancy	138	37.8
Positive at birth	11	3
No positive test recorded	216	59.2
Onset of labor		
Spontaneous	233	63.8
Induction	132	36.2
Birth as expected, partner		
Yes	261	71.5
In vitro fertilisation		
Yes	17	4.7
Missing	44	12.1
Complicated birth*		
Yes	106	29
* Postpartum hemorrhage* > *1000 ml*		
Yes	31	8.5
Missing	< 5	
* Manual placenta removal*		
Yes	14	3.8
Mode of birth		
Vaginal non instrumental birth	309	84.7
*Vaginal instrumental birth*	23	6.3
*Emergency cesarean section*	33	9
* Apgar* < *7 at 5 min*		
Yes	7	1.9
Missing	< 5	
* Preterm birth* ≤ *36* + *6*		
Yes	21	5.8
* Immediate NICU admission*		
Yes	26	7.1

Sample size (n). *Complicated birth is defined as the occurrence of one or more of the following obstetric or neonatal outcomes: postpartum hemorrhage > 1000 ml, manual removal of the placenta, vaginal instrumental birth, acute caesarean section, Apgar score < 7 at 5 min, preterm birth (≤ 36 + 6 gestational weeks), or immediate admission to a neonatal intensive care unit (NICU)

*%* percentage, *SOC* Sense of Coherence

### Inclusion of covariates in the analytical models

For research question two, the analysis examined associations between multiple explanatory variables and the outcome in order to identify statistically significant relationships. As this phase was exploratory in nature, no adjustments were made for mediators, moderators or confounders.

For research question three, potential mediators, moderators and confounders identified during the analysis of research question two were included as covariates in the adjusted models to account for their possible influence on the observed associations. The dimension ‘Worry’ included *complicated birth* as covariate. For ‘Information’ and ‘Acceptance’, dissatisfaction with antenatal preparations was included. No additional variables were included for emotional support, as no significant associations were identified.

### Statistical analysis

The data were analysed using R 4.4.3 [33] and Rstudio 2024.12.1.563 [33], along with the tidyverse [34], readxl [35], and car [36] packages. The significance level for all analyses was set at < 0.05 (two-sided). Missing data were treated as a separate category in all analyses to avoid listwise deletion. Missing data were present for two variables: educational level of the partner (*n* = 2) and SOC (*n* = 178). SOC missingness was substantial because the SOC questionnaire was administered at a different time point than the FTFQ, and responses collected after childbirth (*n* = 95) were excluded. Comparisons between participants with and without SOC data showed minimal systematic differences, except for small variations in IVF treatment and year of birth. This pattern indicates that SOC missingness was primarily procedural.

To investigate partners’ childbirth experiences during the COVID-19 pandemic, the mean and standard deviation for each dimension of the FTFQ were calculated. A comparison between participants who answered ‘yes’ or ‘no’ to ‘birth as expected’ was conducted using t-tests for each dimension of the FTFQ. Hierarchical multiple linear regression was used to investigate which of the variables of sociodemographic determinants, global orientation towards life, and obstetric determinants could explain partners’ variation in birth experience for each dimension of the FTFQ. All models for this research question were estimated in two steps: (1) partially adjusted models, including the most relevant variables according to theory, and (2) fully adjusted models, including all identified confounders including COVID-19 specific variables. One participant answered < 50% of the questions for the dimension ‘emotional support’ and was thereby excluded from that dimension in all analyses.

The association between anxiety and/or depression during pregnancy and partners’ childbirth experience was analysed using multiple linear regression for each dimension, both unadjusted and adjusted for the significant variables from the previous analysis. As a sensitivity analysis, the model was run separately for anxiety and depression. As an additional sensitivity analysis, all regression models were re-run including only male first-time partners, corresponding to the population for which the FTFQ was originally developed.

An attrition analysis was conducted to compare participants in our study with those who were eligible but did not complete the FTFQ, using the chi-square test.

## Results

**Fig. 1:**
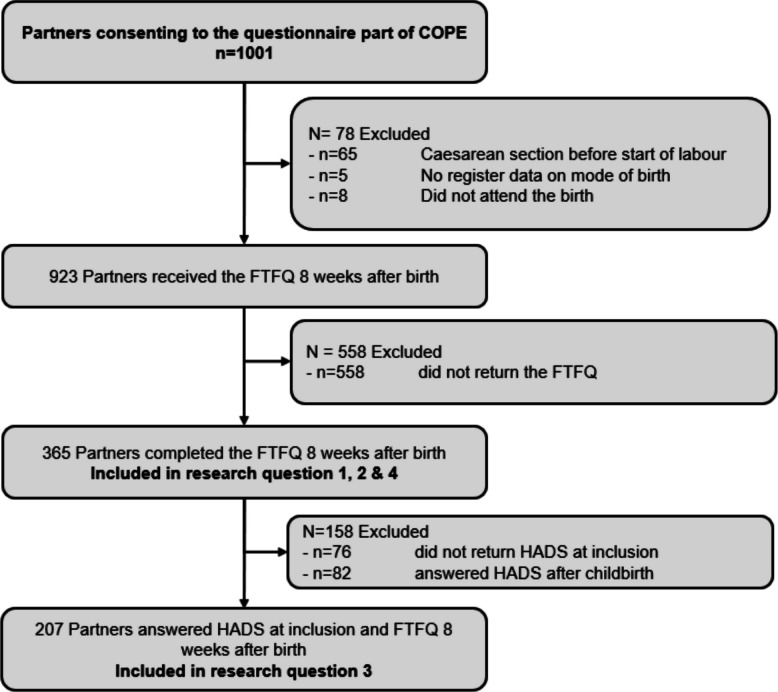
Flow chart of the study population and exclusion process

In total, *n* = 1001 partners consented to participate in the questionnaire component of the COPE study. Of these, *n* = 78 were excluded due to not meeting the inclusion criteria for the current study or due to missing data on mode of birth in the Swedish Pregnancy Register. Among the remaining *n* = 923 participants, *n* = 558 did not complete the FTFQ (15), resulting in a final sample of *n* = 365 participants included in the analysis of research questions 1, 2, and 4 (39.5% response rate). For research question 3, an additional *n* = 158 participants were excluded due to missing HADS data prior to childbirth (24.6% response rate). An overview of the study population and exclusion process is presented in Fig. 1 (flow chart).

Demographic and baseline characteristics of the study population are presented in Table 1. The majority were male (98.4%), first-time parents (54.5%), and living with the birthing woman (94.2%). Most partners were between 31 and 40 years of age (65.0%), and 73.2% had a university-level education. Approximately one in eight partners (13.4%) reported a low SOC, and 29% of births were classified as a *complicated birth.*

Partners’ experiences of childbirth during the COVID-19 pandemic are presented in Table 2. The mean scores (± SD) for each dimension of the FTFQ were as follows: ‘Worry’ 2.11 ± 0.69, ‘Information’ 1.66 ± 0.53, ‘Acceptance’ 1.28 ± 0.42, and ‘Emotional support’ 2.07 ± 0.71. In total, 28.5% of participants responded “no” to the item *“Did you experience the childbirth as you had expected?”,* categorized into the variable ‘birth as expected’*.* This subgroup reported significantly higher scores for the ‘Worry’, ‘Information’, and ‘Acceptance’ dimensions.

**Table 2:** Partners’ experiences of childbirth during the COVID-19 pandemic, as measured by the Fathers for the First Time Questionnaire (FTFQ), among 365 participants in the COPE study

	Total		Birth as expected					
	***N* = 365**		**No** *n* = 104 28.5%		**Yes** *n* = 261 71.5%			
**Coefficients**	**Mean**	**SD**	**Mean**	**SD**	**Mean**	**SD**	**t-test**	***p-value***
Worry	2.1	0.7	2.5	0.7	2.0	0.6	6.48	**< 0.001**
Information	1.7	0.5	1.9	0.5	1.6	0.5	4.62	**< 0.001**
Acceptance	1.3	0.4	1.4	0.5	1.2	0.4	3.14	**0.002**
Emotional support	2.1	0.7	2.1	0.6	2.1	0.7	0.60	0.55

*p* < 0.05 is marked in bold

*Birth as expected* was measured using the self-reported item: “Did you experience the childbirth as you had expected?”, with a binary response option (yes/no)

*SD* standard deviation, *N* total population size, *n* sample size, *%* percentage, *t* t-test statistic, *p-value* significance level

The association of the different sociodemographic and obstetric determinants, as well as variables for global orientation towards life, with partners’ experiences of childbirth during the COVID-19 pandemic is presented in Table 3. The composite exposure variable *complicated birth* was statistically significantly associated with higher levels of ‘Worry’. This association remained significant in both the partially and fully adjusted models. A sensitivity analysis on the individual components of *complicated birth* indicated that manual placenta removal and emergency caesarean section were significantly associated with increased ‘Worry’ (see Supplementary table ‘S1’). Not being satisfied with antenatal preparations was significant for the partly adjusted, but not fully adjusted, model. However, due to satisfaction with preparations not being consistent across both models, along with it only being just significant (*p* = 0.05), it was decided not to include this variable as a covariate in the analyses on depression and/or anxiety for ‘Worry’.

**Table 3:** Associations between sociodemographic determinants, global orientation toward life, obstetric determinants, and partners’ self-reported childbirth experience, *n* = 365, from the COPE study

**Worry**	**Step 1**		**Step 2**	
Coefficients	**B (CI 95%)**	***p-value***	**B (CI 95%)**	***p-value***
First time parent, partner	0.19 (−0.11–0.49)	0.21	0.14 (−0.16 – 0.45)	0.36
Primipara birthing woman	0.07 (−0.23–0.37)	0.64	0.12 (−0.19 – 0.42)	0.45
Not satisfied with preparations	0.19 (0.00–0.37)	**0.046**	0.18 (−0.00 – 0.37)	0.052
Low SOC	0.12 (−0.10–0.33)	0.28	0.10 (−0.12 – 0.31)	0.39
Not replied to SOC	0.15 (0.00–0.29)	**0.048**	0.13 (−0.01 – 0.28)	0.08
Induction	0.10 (−0.04–0.24)	0.16	0.10 (−0.045– 0.24)	0.18
*Complicated birth*	0.34 (0.19–0.49)	**< 0.001**	0.35 (0.20–0.50)	**< 0.001**
Child born 2021			0.00 (−0.17 – 0.18)	0.99
Child born 2022			0.20 (−0.11 – 0.50)	0.20
Not vaccinated at birth, birthing woman			0.18 (−0.04 – 0.40)	0.11
Covid during pregnancy, birthing woman			−0.02 (−0.17 – 0.12)	0.78
Covid during birth, birthing woman			0.01 (−0.39 – 0.41)	0.96
Partner education at university level			−0.08 (−0.23 – 0.08)	0.32
Partner education at university level missing			0.37 (−0.56 – 1.30)	0.43

**Information**	**Step 1**		**Step 2**	
Coefficients	**B (CI 95%)**	***p-value***	**B (CI 95%)**	***p-value***
First time parent, partner	0.13 (−0.11–0.36)	0.28	0.09 (−0.15 – 0.33)	0.46
Primipara, birthing woman	0.02 (−0.21–0.25)	0.87	0.06 (−0.18 – 0.30)	0.64
Not satisfied with preparations	0.35 (0.21–0.50)	**< 0.001**	0.34 (0.19–0.48)	**< 0.001**
Low SOC	0.13 (−0.04–0.30)	0.13	0.11 (−0.06 – 0.28)	0.20
Not replied to SOC	0.04 (−0.07–0.16)	0.454	0.03 (−0.08 – 0.15)	0.58
Induction	−0.06 (−0.17–0.05)	0.32	−0.08 (−0.19 – 0.04)	0.19
*Complicated birth*	0.03 (−0.09–0.15)	0.60	0.05 (−0.07 – 0.17)	0.44
Child born 2021			−0.03 (−0.17 – 0.11)	0.67
Child born 2022			−0.03 (−0.26 – 0.21)	0.83
Not vaccinated at birth, birthing woman			0.11 (−0.06 – 0.28)	0.20
Covid during pregnancy			0.03 (−0.08 – 0.15)	0.57
Covid during birth			0.09 (−0.22 – 0.41)	0.55
Partner education at university level			−0.11 (−0.24 – 0.01)	0.06
Partner education at university level missing			0.47 (−0.25 – 1.20)	0.19

**Acceptance**	**Step 1**		**Step 2**	
Coefficients	**B (CI 95%)**	***p-value***	**B (CI 95%)**	***p-value***
First time parent, partner	0.05 (−0.14–0.24)	0.61	0.07 (−0.13 – 0.27)	0.48
Primipara birthing woman	−0.01 (−0.21–0.18)	0.89	−0.03 (−0.23 – 0.17)	0.79
Not satisfied with preparations	0.14 (0.03–0.26)	**0.02**	0.14 (0.02–0.26)	**0.02**
Low SOC	0.11 (−0.03–0.25)	0.12	0.12 (−0.02 – 0.26)	0.094
Not replied to SOC	0.02 (−0.07–0.12)	0.61	0.02 (−0.07 – 0.12)	0.66
Induction	−0.03 (−0.12–0.06)	0.51	−0.03 (−0.13 – 0.06)	0.48
*Complicated birth*	0.05 (−0.05–0.14)	0.37	0.04 (−0.06 – 0.14)	0.40
Child born 2021			−0.04 (−0.15 – 0.07)	0.50
Child born 2022			−0.03 (−0.23 – 0.17)	0.76
Not vaccinated at birth, birthing woman			0.02 (−0.12 – 0.16)	0.79
Covid during pregnancy, birthing woman			0.04 (−0.06 – 0.13)	0.42
Covid during birth, birthing woman			−0.09 (−0.35 – 0.17)	0.49
Partner education at university level			0.01 (−0.08 – 0.12)	0.75
Partner education at university level missing			−0.27 (−0.87 – 0.34)	0.39

**Emotional support**	**Step 1**		**Step 2**	
Coefficients	**B (CI 95%)**	***p-value***	**B (CI 95%)**	***p-value***
First time parent, partner	−0.20 (−0.52–0.13)	0.23	−0.15 (−0.48 – 0.18)	0.38
Primipara, birthing woman	−0.07 (−0.39–0.26)	0.69	−0.11 (−0.44 – 0.22)	0.51
Not satisfied with preparations	−0.02 (−0.18–0.22)	0.84	0.02 (−0.18 – 0.22)	0.86
Low SOC	0.14 (−0.19–0.28)	0.72	0.06 (−0.17 – 0.30)	0.60
Not replied to SOC	0.02 (−0.14–0.18)	0.82	−0.01 (−0.17 – 0.15)	0.94
Induction	−0.06 (−0.21–0.10)	0.47	−0.07 (−0.22 – 0.09)	0.39
*Complicated birth*	0.01 (−0.16–0.17)	0.95	0.01 (−0.16 – 0.18)	0.91
Child born 2021			−0.13 (−0.32 – 0.06)	0.17
Child born 2022			−0.01 (−0.34 – 0.32)	0.96
Not vaccinated at birth, birthing woman			0.04 (−0.19 – 0.28)	0.72
Covid during pregnancy, birthing woman			0.08 (−0.08 – 0.24)	0.33
Covid during birth, birthing woman			0.22 (−0.21 – 0.65)	0.32
Partner education at university level			0.13 (−0.03 – 0.30)	0.12
Partner education at university level missing			0.10 (−0.91 – 1.11)	0.85

Childbirth experience was measured using the Fathers for the First Time Questionnaire (FTFQ). *p* < 0.05 is marked in bold

*B* unstandardised regression coefficient, *CI* confidence interval, *p-value* significance level, *SOC* Sense of Coherence

Partners who reported being ‘not satisfied with birth preparations’ had significantly higher scores on the ‘Information’ and ‘Acceptance’ dimensions compared to those who were satisfied with their preparations. This association remained statistically significant in both the partially and fully adjusted models for both dimensions. No variables reached statistical significance for the dimension ‘Emotional support’.

The regression analyses for the four dimensions of the FTFQ demonstrated varying degrees of explanatory power. For the dimension ‘Worry’, both the partially adjusted (R^2^_adj_ = 0.13, *p* < 0.001) and fully adjusted (R^2^_adj_ = 0.12, *p* < 0.001) models were statistically significant, indicating a moderate explanatory contribution. The models for ‘Information’ also showed statistically significant results, explaining 8.5% (R^2^_adj_ = 0.09, *p* < 0.001) and 9.3% (R^2^_adj_ = 0.09, *p* < 0.001) of the variance in the partially and fully adjusted models, respectively. For ‘Acceptance’, neither the partially adjusted model (R^2^_adj_ = 0.01, *p* = 0.09), nor the fully adjusted model was statistically significant (R^2^_adj_ = 0.001, *p* = 0.37) and both had limited explanatory value.

The associations between partners’ anxiety and/or depression during pregnancy and their childbirth experiences are presented in Table 4. Anxiety and/or depression were significantly associated with higher scores for ‘Worry’ and ‘Information’ in both unadjusted and adjusted analyses, but not for ‘Acceptance’ and ‘Emotional support’. All confounders remained significant in the adjusted models. When anxiety and depression were analysed separately, results remained mainly unchanged (see Supplementary Tables S2–S3).

**Table 4:** Associations between anxiety and/or depression and partners’ self-reported childbirth experience, *n* = 207, from the COPE study

	**Unadjusted**		**Adjusted**	
Worry	**B (CI 95%)**	***p-value***	**B (CI 95%)**	***p-value***
Anxiety and/or Depression	0.34 (0.12–0.56)	**0.003**	0.31 (0.10–0.52)	**0.004**
*Complicated birth*			0.43 (0.24–0.62)	**< 0.001**
Information				
Anxiety and/or depression	0.27 (0.11–0.44)	**0.001**	0.20 (0.04–0.36)	**0.014**
Not satisfied with preparations			0.42 (0.24–0.60)	**< 0.001**
Acceptance				
Anxiety and/or depression	0.10 (−0.05–0.24)	0.18	0.06 (−0.09 – 0.20)	0.437
Not satisfied with preparations			0.23 (0.07–0.39)	**0.005**
Emotional support				
Anxiety and/or depression	0.00 (−0.22–0.22)	0.97		

Anxiety and depression were measured using the Hospital Anxiety and Depression Scale (HADS). Childbirth experience was assessed with the Fathers for the First Time Questionnaire (FTFQ). *p* < 0.05 is marked in bold. Since no variables reached statistical significance for the dimension “emotional support” in the previous analysis, no adjustments were included in Step 2

*B* unstandardised regression coefficient, *CI* confidence interval, *p-value* significance level

The regression models demonstrated varying degrees of explanatory power across the four dimensions of the FTFQ. For the dimension ‘Worry’, the partially adjusted model explained 3.8% of the variance (R^2^_adj_ = 0.04, *p* = 0.003), while the fully adjusted model accounted for 11.6% (R^2^_adj_ = 0.12, *p* < 0.001). Regarding ‘Information’, the partially adjusted model yielded an R^2^_adj_ of 0.05 (*p* = 0.001), and the fully adjusted model explained 12.8% of the variance (R^2^_adj_ = 0.103 *p* < 0.001). For ‘Acceptance’, the partially adjusted model showed limited explanatory value (R^2^_adj_ = 0.004, *p* = 0.182), whereas the fully adjusted model improved modestly to 3.8% (R^2^_adj_ = 0.04, *p* = 0.007). Finally, for ‘Emotional support’, the model was not statistically significant, with a negative R^2^_adj_ of −0.004 and *p* = 0.970, indicating no meaningful explanatory contribution.

In sensitivity analyses restricted to first-time male partners, the overall pattern of associations remained largely similar; however, the associations between antenatal anxiety and/or depression and the ‘Information’ dimension, as well as between birth complications and ‘Worry’, were attenuated and no longer reached statistical significance. These changes coincided with a substantially reduced sample size. No significant differences were found between partners who answered the FTFQ and those eligible but who did not complete the questionnaire (see Supplementary Table S5).

## Discussion

To the best of our knowledge, this is the largest cohort study exploring partners self-reported childbirth experience during the COVID-19 pandemic. We found that ‘Worry’ was the most negatively rated dimension, with higher FTFQ scores indicating a more negative childbirth experience. ‘Acceptance’ received the lowest (most positive) scores, followed by ‘Information’ and ‘Emotional support’. Partners who did not experience the ‘birth as expected’ reported significantly higher scores on the dimensions of ‘Worry’, ‘Information’, and ‘Acceptance’. A *complicated birth* was associated with increased ‘Worry’, while ‘dissatisfaction with antenatal preparations’ was linked to higher scores on ‘Information’ and ‘Acceptance’. No variables were significantly associated with ‘Emotional support’. Partners with symptoms of anxiety and/or depression during pregnancy reported higher scores for ‘Worry’ and ‘Information’.

Few studies have explored partners’ experiences of childbirth during the pandemic or the association of prenatal depression and/or anxiety with childbirth experience. Our findings contribute valuable insights and may assist healthcare professionals in developing more inclusive approaches to supporting partners during pregnancy and childbirth. In an Australian cohort (*n* = 92), 62% of the surveyed partners stated that ‘compared with my expectations, some of our experiences turned out better than I thought they might during the COVID-19 pandemic’ [37]. Protocols regarding partner presence during and after childbirth varied across Sweden during the COVID-19 pandemic, some regions allowed partners to be present throughout both labour and the postpartum period, while others restricted their presence to the labour phase only. While the extent of partner presence varied across regions, qualitative findings from three Swedish studies indicate that partners felt excluded, unprepared and worried [14, 38, 39]. In our study, partners who did not experience ‘birth as expected’ reported significantly higher levels of ‘Worry’. This finding is consistent with the well-described “expectation–reality gap,” in which a mismatch between anticipated and actual events intensifies feelings of worry, loss of control and emotional strain, as shown in previous qualitative research [40]. *Complicated births*, such as postpartum hemorrhage > 1000 ml, emergency cesarean section, or manual placenta removal, were associated with increased ‘Worry’. A narrative systematic review found that fathers reported feeling ill-prepared for complications, as the information they had received beforehand focused primarily on normal labour and birth. This lack of preparation led to feelings of helplessness and worry when complications arose [16]. Childbirth has previously been described as an emotional rollercoaster, particularly in the context of *complicated births* [41]. Such unpredictability may intensify partners’ experience of anxiety and sense of exclusion [42]. It is, however, important to note that partners’ perceptions of childbirth may extend beyond clinical indicators, as experiences of fear, helplessness or loss of control can be interpreted as traumatic even in the absence of medical complications [40].

Symptoms of anxiety and/or depression during pregnancy were associated with increased ‘Worry’. A similar association has been found in birthing women where prenatal anxiety had a negative effect on birth experience [43]. However, we were unable to identify any studies that specifically examined partners’ symptoms of anxiety and depression in relation to their experience of childbirth. It is encouraging that antenatal anxiety and depression among partners is receiving growing recognition. A recent systematic review and meta-analysis identified mismatched expectations regarding pregnancy and childbirth as risk factors for paternal perinatal anxiety and depression [44]. Furthermore, antenatal anxiety and depression in partners have been linked to subsequent postnatal mental health difficulties [45]. Postnatal depression in partners may influence their parenting approach and, in turn, potentially affect the child’s behavioural and social development in adverse ways [46].

Our study found that partners who did not experience the ‘birth as expected’, or who reported symptoms of depression and/or anxiety during pregnancy, were more likely to report higher (i.e., worse) scores on the ‘Information’ dimension. According to the original validation of the FTFQ, the Information subscale captures four key aspects of partners’ perceived preparedness and informational support during childbirth: whether they felt adequately informed about the birth process, whether the information was relevant and sufficient, how well they understood what was happening in the labour room, and whether they felt included through ongoing updates during the birth [20]. Higher scores on this dimension in our study therefore likely reflect gaps in these areas, which may have been amplified by rapidly changing guidelines and communication constraints during the pandemic. Qualitative studies have described how limited support and unpredictable changes contributed to partners’ feelings of anxiety and helplessness, despite efforts to prepare. Swedish studies conducted during the pandemic show that partners often felt insufficiently informed and had limited opportunities to ask questions or receive individualised guidance antenatally, which may have contributed to increased uncertainty and worry [14, 38, 39]. Even those aware of potential complications reported feeling unprepared for the emotional and practical realities of childbirth [42]. Effective communication from healthcare professionals was described as a factor that could buffer distress and support a more positive childbirth experience [42, 47]. These findings underscore the importance not only of providing information, but also of how that information is communicated, particularly during periods of heightened stress. Rather than focusing solely on increasing the quantity of information, partners may benefit more from support aimed at strengthening emotional resilience and coping strategies. In clinical practice, a flexible and supportive approach may be more effective than simply expanding informational resources. It may also be advisable to individualise communication for partners with a history of anxiety or depression already during the antenatal period, in order to help mitigate the risk of traumatic experiences.

Partners who did not experience childbirth as expected also reported higher scores on the ‘Acceptance’ dimension. However, the explanatory power of our model was limited. No variables accounted for variance in ‘Emotional support’. In the historical validation of the FTFQ, both ‘Emotional support’ and ‘Acceptance’ failed to meet conventional reliability standards for group comparisons [20], suggesting that the FTFQ may not adequately capture these dimensions.

No clear association was found between specific COVID-19-related factors, such as year of birth, vaccination status or SARS-CoV-2 infection, and partners’ experiences of childbirth. Previous findings from Sweden indicate that partners experienced increased worry during the early stages of the pandemic and reported elevated anxiety regarding the health of their child, their pregnant partner, and themselves. These heightened levels of worry appeared to remain relatively stable throughout the course of the pandemic [48]. This sustained psychological impact may help explain the lack of clear differences in partners’ childbirth experiences based on specific COVID-19 exposure variables. It is possible that general uncertainty and prolonged stress had a greater influence than these individual factors. However, this interpretation should be made with caution, given the limited number of observations within each COVID-19-related variable. Reviews of maternity care during COVID-19 describe how reduced support, altered care pathways and rapidly changing restrictions contributed to heightened anxiety, feelings of isolation and reduced emotional safety among expectant parents [11, 49]. Systematic reviews of natural and human-made disasters similarly report elevated levels of maternal anxiety, depression and post-traumatic stress following crises, particularly when access to support or stable care environments is disrupted [50, 51]. Taken together, this literature suggests that the heightened worry observed among partners in our study reflects broader psychological responses that arise when childbirth occurs under crisis conditions.

Although no formal comparison was undertaken, the mean levels observed in our study fall within the general range reported in earlier Swedish research using the FTFQ among first-time fathers in 2008, prior to the pandemic [20]. This descriptive observation provides contextual background but does not permit any inference of similarity across cohorts. As partner childbirth experiences have not been measured systematically over time in Sweden, it remains unclear how these experiences have evolved across different periods or healthcare contexts.

Among partners born outside Sweden (10.7%), higher levels of worry were observed. This pattern is consistent with earlier Swedish research using the FTFQ, where partners born outside Sweden also reported higher levels of worry [20]. Similar trends have been reported previously, with non-Swedish-born partners more likely to report fear of childbirth, a factor associated with more negative birth experiences [52]. Broader evidence from paternal perinatal mental health research similarly shows that fathers from ethnic minority groups, low-income backgrounds and those with previous trauma histories are at increased risk of anxiety and psychological distress during the perinatal period [53]. These findings suggest that foreign-born partners may constitute a particularly vulnerable group in need of tailored support. Language barriers and communication challenges may contribute to increased worry and feelings of exclusion, especially during the pandemic and in settings where personal protective equipment was used [54]. Clinically, acknowledging and addressing these disparities may be important for improving childbirth experiences for all partners.

## Strength and limitations

The current study has several strengths. This cross-sectional prospective cohort study utilised data obtained from national health and quality registers as well as validated questionnaires, the latter enhancing the reliability of self-reported measures. The Swedish quality registers are internationally recognised for their comprehensive coverage and high data quality, which strengthens the overall validity of the findings [55]. While it is unknown how many partners were initially invited (due to pandemic-related restrictions), the participation of most maternity units across Sweden strengthens generalisability. Statistically significant findings indicate an adequate sample size, with regression models explaining 0.9–13.8% of the variance. Sensitivity analyses restricted to first-time male partners yielded largely similar patterns, although some associations were attenuated, likely reflecting reduced statistical power due to smaller sample sizes. Future studies are needed to explore additional determinants influencing partners’ experiences. The main limitation of this study is the low response rate: 36.5% for research questions 1, 2 and 4, and 24.6% for research question 3. This was expected, as recruitment relied on birthing women to invite their partners to participate. While missing data are common in survey-based research, our regression analyses suggest that the results are robust. Attrition analysis showed no significant differences between completers and dropouts, including in relation to experiences of *complicated birth*, supporting the reliability of the findings. A further limitation concerns missing data on the SOC scale. SOC was collected at a different time point than the childbirth questionnaire, resulting in substantial procedural missingness. Because the missingness pattern was mainly unrelated to background characteristics and no strong predictors of SOC were available, multiple imputation or full information maximum likelihood would have been unlikely to produce reliable estimates. We therefore treated missing SOC as a separate category, which preserves sample size but does not fully account for uncertainty related to missingness. Future studies with richer auxiliary data may be better positioned to apply more advanced missing data techniques. Generalisability to other high income settings may be limited due to an overrepresentation of highly educated and Swedish-born participants compared to the broader Swedish male population. Finally, the FTFQ was originally validated in first-time fathers, and its psychometric properties have not been established for partners with previous childbirth experience Although internal consistency estimates in our sample were largely comparable to those reported in the original validation study, modest reliability observed for the Information and Acceptance subscales, particularly among experienced partners, represents a methodological limitation and should be considered when interpreting findings related to these dimensions. Similarly, although the FTFQ was applied across cis-gender and gender-diverse non-birthing partners, the instrument has not been validated for LGBTQ + partners. Research suggests that LGBTQ + parents may face additional challenges related to recognition, support and heteronormative assumptions in maternity care, which may influence their childbirth experiences [56]. Future validation work is therefore warranted.

## Conclusion

This study highlights several key factors associated with partners’ self-reported childbirth experience during the COVID-19 pandemic, as measured by the FTFQ. Not experiencing the birth as expected and being exposed to a *complicated birth* were both associated with higher scores on the dimensions ‘Worry’, Information’, and ‘Acceptance’, indicating a more negative childbirth experience. Similarly, symptoms of anxiety and/or depression during pregnancy were linked to increased ‘Worry’, while dissatisfaction with antenatal preparations was associated with higher scores on both ‘Information’ and ‘Acceptance’. No variables were significantly associated with the dimension ‘Emotional support’. These findings underscore the importance of identifying psychological vulnerability and unmet informational needs in partners during pregnancy, and of fostering communication strategies that promote inclusion and emotional safety in the childbirth setting.

## Supplementary Information

Supplementary Material 1.

## Data Availability

The data that support the findings of this study are available from Statistics Sweden (SCB) and the Swedish National Board of Health and Welfare (Socialstyrelsen), but restrictions apply to the availability of these data, which were used under license for the current study and are not publicly available. No individual-level data can be shared due to data protection and privacy legislation. The use of microdata from the national registers is regulated by Swedish law and the General Data Protection Regulation (GDPR). However, data are available from the authors upon reasonable request and with permission of Statistics Sweden and the National Board of Health and Welfare.

## Abbreviations

COPE: COVID-19 during Pregnancy and Early Childhood
FTFQ: Fathers for the First Time Questionnaire
HADS: Hospital Anxiety and Depression Scale
SOC: Sense of Coherence scale
