# Supplementary material for: Partners’ childbirth experiences during the COVID-19 pandemic – findings from the Swedish COPE prospective cohort study
Source: BMC Pregnancy Childbirth. 2026 Feb 27;26:223. doi: 10.1186/s12884-026-08862-3 (PMC12955138; doi:10.1186/s12884-026-08862-3)
Supplement: Supplementary file 1 — Supplementary Material 1. [file 12884_2026_8862_MOESM1_ESM.docx]

# Supplementary material

*Table S1. Sensitivity analysis with individual variables that comprise the composite variable Complicated birth, adjusted for all other variables in the partly adjusted model.*

|  | **B (CI 95%)** | ***p-value*** |
| --- | --- | --- |
| **Worry** |  |  |
| Postpartum hemorrhage >1000 ml | -0.06 (-0.33 – 0.22) | 0.67 |
| Postpartum hemorrhage missing | -0.40 (-1.06 – 0.25) | 0.22 |
| Manual placenta removal | 0.46 (0.07 – 0.86) | **0.02** |
| Vaginal instrumental birth | 0.23 (-0.05 – 0.51) | 0.10 |
| Emergency cesarean section | 0.34 (0.09 – 0.59) | **0.008** |
| Apgar <7 at 5 min | 0.28 (-0.27 – 0.83) | 0.32 |
| Apgar missing | 0.76 (0.02 – 1.51) | **0.04** |
| Preterm birth ≤36+6 | 0.07 (-0.24 – 0.37) | 0.65 |
| Immediate NICU admission | 0.26 (-0.04 – 0.55) | 0.09 |
| **Information** |  |  |
| Postpartum hemorrhage >1000 ml | -0.03 (-0.24 – 0.19) | 0.81 |
| Postpartum hemorrhage missing | 0.26 (-0.26 – 0.77) | 0.32 |
| Manual placenta removal | 0.04 (-0.27 – 0.35) | 0.78 |
| Vaginal instrumental birth | -0.11 (-0.33 – 0.11) | 0.30 |
| Emergency cesarean section | 0.17 (-0.03 – 0.36) | 0.09 |
| Apgar <7 at 5 min | 0.18 (-0.25 – 0.61) | 0.41 |
| Apgar missing | 0.16 (-0.42 – 0.75) | 0.58 |
| Preterm birth ≤36+6 | 0.11 (-0.13 – 0.35) | 0.36 |
| Immediate NICU admission | 0.02 (-0.22 – 0.25) | 0.90 |
| **Acceptance** |  |  |
| Postpartum hemorrhage >1000 ml | -0.02 (-0.20 – 0.16) | 0.82 |
| Postpartum hemorrhage missing | -0.03 (-0.40 – 0.46) | 0.89 |
| Manual placenta removal | 0.05 (-0.21 – 0.31) | 0.71 |
| Vaginal instrumental birth | 0.03 (-0.15 – 0.22) | 0.71 |
| Emergency cesarean section | 0.08 (-0.09 – 0.24) | 0.35 |
| Apgar <7 at 5 min | 0.31 (-0.05 – 0.67) | 0.08 |
| Apgar missing | -0.01 (-0.50 – 0.48) | 0.96 |
| Preterm birth ≤36+6 | 0.10 (-0.10 – 0.30) | 0.33 |
| Immediate NICU admission | -0.13 (-0.33 – 0.07) | 0.18 |
| **Emotional support** |  |  |
| Postpartum hemorrhage >1000 ml | 0.11 (-0.19 – 0.42) | 0.45 |
| Postpartum hemorrhage missing | 0.30 (-0.42 – 1.01) | 0.41 |
| Manual placenta removal | -0.08 (-0.51 – 0.35) | 0.70 |
| Vaginal instrumental birth | 0.23 (-0.07 – 0.54) | 0.13 |
| Emergency cesarean section | -0.13 (-0.41 – 0.14) | 0.32 |
| Apgar <7 at 5 min | 0.10 (-0.50 – 0.70) | 0.74 |
| Apgar missing | 0.47 (-0.52 – 1.47) | 0.35 |
| Preterm birth ≤36+6 | -0.09 (-0.43 – 0.25) | 0.59 |
| Immediate NICU admission | 0.05 (-0.28 – 0.38) | 0.76 |

B Unstandardized coefficients, CI Confidence interval, p-value - Significance, NICU Neonatal Intensive Care Unit, p <0.05 is marked in bold 
The model was adjusted for: First time parent, partner; Primipara, birthing woman; Not satisfied with birth preparations: Low SOC; Not replied to SOC; Induction of labour.

*Table S2. Associations between mild/significant anxiety and partners’ self-reported childbirth experience, n = 207, from the COPE study*

|  | **Unadjusted** | | | | **Adjusted** | | | |
| --- | --- | --- | --- | --- | --- | --- | --- | --- |
|  | **B** |  |  | ***p-value*** | **B** |  |  | ***p-value*** |
| **Worry** |  |  |  |  |  |  |  |  |
| Anxiety Mild/Significant | 0.32 (0.10 – 0.54) |  |  | **0.005** | 0.29 (0.08 – 0.50) |  |  | **0.008** |
| *Complicated birth* |  |  |  |  | 0.43 (0.23 – 0.62) |  |  | **<0.001** |
| **Information** |  |  |  |  |  |  |  |  |
| Anxiety Mild/Significant | 0.26 (0.09 – 0.42) |  |  | **0.003** | 0.18 (0.02 – 0.34) |  |  | **0.03** |
| Not satisfied with preparations |  |  |  |  | 0.42 (0.24 – 0.60) |  |  | **<0.001** |
| **Acceptance** |  |  |  |  |  |  |  |  |
| Anxiety Mild/Significant | 0.10 (-0.04 – 0.25) |  |  | 0.148 | 0.06 (-0.08 – 0.21) |  |  | 0.381 |
| Not satisfied with preparations |  |  |  |  | 0.23 (0.07 – 0.39) |  |  | **0.005** |
| **Emotional support*** |  |  |  |  |  |  |  |  |
| Anxiety Mild/Significant | 0.02 (-0.20 – 0.24) |  |  | 0.861 |  |  |  |  |

B Unstandardized coefficients, CI Confidence interval, p-value: Significance, p<0.05 is marked in bold

*As no variables showed statistical significance for emotional support in the previous analysis, they were not included as adjustments in Step 2

*Table S3. Associations between mild/significant depression and partners’ self-reported childbirth experience, n = 207, from the COPE study*

| **Worry** | **Step 1 (Unadjusted)** | | | | **Step 2 (Adjusted)** | | | |
| --- | --- | --- | --- | --- | --- | --- | --- | --- |
| Coefficients | **B (CI 95%)** | **β** | **t** | ***p-value*** | **B (CI 95%)** | **β** | **t** | ***p-value*** |
| Depression Mild/Significant | 0.39 (0.04 – 0.75) | 0.153 | 2.21 | **0.028** | 0.33 (-0.01 – 0.67) | 0.128 | 1.92 | 0.056 |
| *Complicated birth* |  |  |  |  | 0.43 (0.23 – 0.63) | 0.285 | 4.29 | **<0.001** |
| **Information** | **Step 1 (Unadjusted)** | | | | **Step 2 (Adjusted)** | | | |
| Coefficients | **B (CI 95%)** | **β** | **t** | ***p-value*** | **B (CI 95%)** | **β** | **t** | ***p-value*** |
| Depression Mild/Significant | 0.36 (0.09 – 0.63) | 0.183 | 2.67 | **0.008** | 0.32 (0.07 – 0.58) | 0.093 | 2.52 | **0.012** |
| Not satisfied with preparations |  |  |  |  | 0.45 (0.27 – 0.63) | 0.265 | 4.96 | **<0.001** |
| **Acceptance** | **Step 1 (Unadjusted)** | | | | **Step 2 (Adjusted)** | | | |
| Coefficients | **B (CI 95%)** | **β** | **t** | ***p-value*** | **B (CI 95%)** | **β** | **t** | ***p-value*** |
| Depression Mild/Significant | 0.12 (-0.10 – 0.35) | 0.074 | 1.06 | 0.290 | 0.10 (-0.12 – 0.32) | 0.051 | 0.91 | 0.367 |
| Not satisfied with preparations |  |  |  |  | 0.24 (0.08 – 0.39) | 0.142 | 3.02 | **0.003** |
| **Emotional support*** | **Step 1 (Unadjusted)** | | | |  | | | |
| Coefficients | **B (CI 95%)** | **β** | **t** | ***p-value*** |  |  |  |  |
| Depression Mild/Significant | 0.10 (-0.25 – 0.45) | 0.038 | 0.55 | 0.585 |  |  |  |  |

B Unstandardized coefficients, CI Confidence interval, β Standardized coefficients, t t-test, p-value - Significance, p<0.05 is marked in bold,

*As no variables showed statistical significance for emotional support in the previous analysis, they were not included as adjustments in Step 2

*Table S4. Attrition analysis for partners who did not complete the Father For the First Time Questionnaire (FTFQ)*

|  | **Completed FTFQ** | | **Did not complete FTFQ** | |  |  | ***p-value*** |
| --- | --- | --- | --- | --- | --- | --- | --- |
| Characteristics | **n = 365** | **%** | **n = 558** | **%** |  |  |  |
| **Parity birthing woman** |  |  |  |  |  |  | 0.18 |
| Primipara | 199 | 54.5 | 278 | 49.8 |  |  |  |
| **Age partner** |  |  |  |  |  |  | 0.36 |
| 21-30 | 97 | 26.6 | 176 | 31.5 |  |  |  |
| 31-40 | 236 | 64.7 | 324 | 58.1 |  |  |  |
| >41 | 30 | 8.4 | 57 | 10.2 |  |  |  |
| **Age birthing woman** |  |  |  |  |  |  | 0.28 |
| 21-30 | 126 | 34.5 | 217 | 38.9 |  |  |  |
| 31-40 | 231 | 63.3 | 324 | 58.1 |  |  |  |
| >41 | 8 | 2.2 | 17 | 3.0 |  |  |  |
| **Gender partner** |  |  |  |  |  |  | 0.25 |
| Male | 359 | 98.4 | 555 | 99.5 |  |  |  |
| **Relationship to birthing woman** |  |  |  |  |  |  | 0.30 |
| Living together | 344 | 94.3 | 519 | 93.0 |  |  |  |
| Single living | <5 |  | <5 |  |  |  |  |
| Other | <5 |  | 10 | 1.8 |  |  |  |
| **Year of childbirth** |  |  |  |  |  |  | 0.13 |
| 2020 | 93 | 25.5 | 161 | 28.9 |  |  |  |
| 2021 | 200 | 54.8 | 268 | 48.0 |  |  |  |
| 2022 | 72 | 19.7 | 129 | 23.1 |  |  |  |
| **Vaccinated at birth, birthing woman** |  |  |  |  |  |  | 0.27 |
| Yes | 114 | 31.2 | 154 | 27.6 |  |  |  |
| **Covid status, birthing woman** |  |  |  |  |  |  | 0.67 |
| Positive during pregnancy | 138 | 37.8 | 205 | 36.7 |  |  |  |
| Positive during birth | 11 | 3.0 | 23 | 4.1 |  |  |  |
| Negative | 216 | 59.2 | 330 | 59.1 |  |  |  |
| **Onset of labor** |  |  |  |  |  |  | 0.17 |
| Spontaneous | 233 | 63.8 | 330 | 59.1 |  |  |  |
| Induction | 132 | 36.16 | 228 | 40.86 |  |  |  |
| **In vitro fertalisation** |  |  |  |  |  |  | 0.13 |
| Yes | 17 | 4.7 | 27 | 4.8 |  |  |  |
| ***Complicated birth*** |  |  |  |  |  |  | 1 |
| Yes | 106 | 29.0 | 163 | 29.2 |  |  |  |
| **Postpartum hemorrhage >1000ml** |  |  |  |  |  |  | 0.16 |
| Yes | 31 | 8.5 | 43 | 7.7 |  |  |  |
| **Manual placenta removal** |  |  |  |  |  |  | 0.26 |
| Yes | 14 | 3.84 | 13 | 2.33 |  |  |  |
| **Mode of birth** |  |  |  |  |  |  | 0.21 |
| Vaginal non instrumental birth | 309 | 84.7 | 447 | 80.1 |  |  |  |
| Vaginal instrumental birth | 23 | 6.3 | 48 | 8.6 |  |  |  |
| Emergency cesarean section | 33 | 9.0 | 63 | 11.3 |  |  |  |
| **Apgar <7 at 5 min** |  |  |  |  |  |  | 0.63 |
| Yes | 7 | 1.9 | 7 | 1.3 |  |  |  |
| **Preterm birth ≤36+6** |  |  |  |  |  |  | 0.48 |
| Yes | 21 | 5.8 | 25 | 4.5 |  |  |  |
| **Immediate NICU admission** |  |  |  |  |  |  | 0.29 |
| Yes | 26 | 7.1 | 29 | 5.2 |  |  |  |

n sample size, % percentage, χ² chi-square, , p-value Significance, SOC Sense of Coherence, *Complicated birth*= One or several of following variables; ‘postpartum hemorrhage >1000 ml’, ‘manual placenta removal’, ‘vaginal instrumental birth’, ‘acute cesarean section’, ‘Apgar score below 7 at 5 min’, ‘preterm birth ≤36+6’, and ‘immediate NICU admission’. NICU Neonatal Intensive Care Unit

*Missing data is excluded since the data is obtained from registers. Partner parity, Level of education, Country of birth, partner, Satisfied with preparations, Type of preparation, Low SOC and Birth as expected is not included in the attrition analysis, as this information was obtained from the FTFQ and that data is thereby missing for individuals who did not complete the FTFQ. 
